# How context can impact clinical trials: a multi-country qualitative case study comparison of diagnostic biomarker test interventions

**DOI:** 10.1186/s13063-019-3215-9

**Published:** 2019-02-08

**Authors:** Marco J. Haenssgen, Nutcha Charoenboon, Nga T. T. Do, Thomas Althaus, Yuzana Khine Zaw, Heiman F. L. Wertheim, Yoel Lubell

**Affiliations:** 1 Centre for Tropical Medicine and Global Health, Nuffield Department of Clinical Medicine, University of Oxford, Old Road Campus, Roosevelt Drive, Oxford, OX3 7FZ UK; 2 CABDyN Complexity Centre, Saïd Business School, University of Oxford, Park End Street, Oxford, OX1 1HP UK; 3 Global Sustainable Development, University of Warwick, Ramphal Building, Coventry, CV4 7AM UK; 4 Mahidol Oxford Tropical Medicine Research Unit, Faculty of Tropical Medicine, Mahidol University, 3/F, 60th Anniversary Chalermprakiat Building, 420/6 Rajvithi Road, Bangkok, 10400 Thailand; 5 Oxford University Clinical Research Unit (OUCRU), National Hospital for Tropical Diseases, 78 Giai Phong Street, Hanoi, Vietnam; 6 Department of Global Health and Development, London School of Hygiene and Tropical Medicine, Keppel Street, London, WC1E 7HT UK; 7 Medical Microbiology Department, Radboudumc, Geert Grooteplein Zuid 10, Nijmegen, 6525 Netherlands

**Keywords:** Intervention implementation, Contextual factors, Qualitative research, Antibiotic prescription, Myanmar, Thailand, Vietnam

## Abstract

**Background:**

Context matters for the successful implementation of medical interventions, but its role remains surprisingly understudied. Against the backdrop of antimicrobial resistance, a global health priority, we investigated the introduction of a rapid diagnostic biomarker test (C-reactive protein, or CRP) to guide antibiotic prescriptions in outpatient settings and asked, “Which factors account for cross-country variations in the effectiveness of CRP biomarker test interventions?”

**Methods:**

We conducted a cross-case comparison of CRP point-of-care test trials across Yangon (Myanmar), Chiang Rai (Thailand), and Hanoi (Vietnam). Cross-sectional qualitative data were originally collected as part of each clinical trial to broaden their evidence base and help explain their respective results. We synthesised these data and developed a large qualitative data set comprising 130 interview and focus group participants (healthcare workers and patients) and nearly one million words worth of transcripts and interview notes. Inductive thematic analysis was used to identify contextual factors and compare them across the three case studies. As clinical trial outcomes, we considered patients’ and healthcare workers’ adherence to the biomarker test results, and patient exclusion to gauge the potential “impact” of CRP point-of-care testing on the population level.

**Results:**

We identified three principal domains of contextual influences on intervention effectiveness. First, perceived risks from infectious diseases influenced the adherence of the clinical users (nurses, doctors). Second, the health system context related to all three intervention outcomes (via the health policy and antibiotic policy environment, and via health system structures and the ensuing utilisation patterns). Third, the demand-side context influenced the patient adherence to CRP point-of-care tests and exclusion from the intervention through variations in local healthcare-seeking behaviours, popular conceptions of illness and medicine, and the resulting utilisation of the health system.

**Conclusions:**

Our study underscored the importance of contextual variation for the interpretation of clinical trial findings. Further research should investigate the range and magnitude of contextual effects on trial outcomes through meta-analyses of large sets of clinical trials. For this to be possible, clinical trials should collect qualitative and quantitative contextual information for instance on their disease, health system, and demand-side environment.

**Trial registration:**

ClinicalTrials.gov, NCT02758821 registered on 3 May 2016 and NCT01918579 registered on 7 August 2013.

**Electronic supplementary material:**

The online version of this article (10.1186/s13063-019-3215-9) contains supplementary material, which is available to authorized users.

## Background

“Context is key” [1] in medical interventions because it “interacts, influences, modifies and facilitates or constrains the intervention and the implementation effort” [2]. Contextual factors involve for instance patient characteristics, the political environment, organisational cultures, or the relationship between patients and healthcare staff [3–5], and we should expect them to influence the full spectrum of simple, pragmatic, and complex clinical trials, considering the extremely diverse health systems, socio-economic settings, and epidemiological environments across and within low-income, middle-income, and high-income countries [3, 5–11]. Researchers call for more research in this area because a better understanding of contextual factors can help to improve the implementation and operation of medical interventions [1, 2, 7].

Despite their importance, contextual factors in clinical interventions are surprisingly under-researched [12, 13]. Statistical analysis can help detect the presence of contextual influences on trial outcomes [7, 14]; qualitative research can shed further light on the nature and underlying mechanisms of these influences [5, 10, 15, 16]. Among the few examples is Reynolds et al. [6], who compiled qualitative data from nine trials of malaria diagnostics and treatment across seven low-income and middle-income countries (LMICs) to study real-life implementation processes (e.g. communication between sub-study teams) and their implications for trial data interpretation.

The present study contributed to this narrow yet essential body of knowledge through a qualitative cross-case comparison of three diagnostic biomarker test trials in Southeast Asia. The objective of this paper was to illuminate how the trial context influenced the implementation and operation of medical interventions. Our definition of context followed Damschroder et al. [4] as: “the set of circumstances or unique factors that surround a particular implementation effort.”

Our study was situated against the backdrop of antimicrobial resistance (AMR). AMR is a global health priority that is feared to cause 10 million deaths annually by 2050 [17], the economic costs of which have been likened to the 2008 global financial crisis with a disproportionate impact on LMICs [18–21]. Yet, AMR is a global problem affecting also high-income countries, as can be seen in a recent case of multi-drug-resistant *Neisseria gonorrhoeae* originating in Southeast Asia that had been detected in the UK [22]. Among the many factors that contribute to AMR is the over-use and misuse of antibiotics especially for non-bacterial infections [19, 20, 23]. Across low-income, middle-income, and high-income countries, such over-use has been attributed partially to antibiotic over-prescription [23–26]. Various interventions are therefore being explored to change prescription patterns [25, 27], among which diagnostic technologies to improve the behaviour of prescribers of antibiotics have received particular attention [28–31].

This study focused on one such diagnostic technology, namely a point-of-care finger-prick blood test for C-reactive protein (CRP). CRP point-of-care tests (POCTs) had been designed to help healthcare staff at the primary-care level to distinguish bacterial from non-bacterial infections [32, 33]. They had been evaluated in high-income countries [34–36], but LMICs are particularly interesting settings for CRP POCTs for two main reasons: (1) the widespread over-use of antibiotics alongside the higher risk of under-treating potentially severe bacterial infections and (2) the frequent absence of state-of-the-art laboratory facilities at the primary-care level [37, 38].

We situated our study within large-scale clinical trials of CRP POCTs in Yangon (Myanmar), Chiang Rai (Thailand), and Hanoi (Vietnam). The purpose of this paper was not to ascertain whether these CRP POCTs met their objectives (which we report elsewhere [39]), but rather to understand how a standardised intervention - implemented with similar trial design and outcome measurement in 20 primary-care sites of three Southeast Asian countries - could yield heterogeneous outcomes. For example, while overall prescriptions of antibiotics decreased in the clinical trials, there were variable and often high degrees of prescribing in patients with a negative test result (i.e. no antibiotic recommended). Healthcare workers (HCWs)’ adherence to a negative test result was 78% on average in Thailand, 81% in Myanmar, and 65% in Vietnam. In the case of Vietnam, these rates varied from 29% to 96% across the participating sites (based on reported immediate prescriptions [39]).

We collected a wealth of qualitative data alongside the clinical trials to contextualise such heterogeneous study outcomes. We drew in this paper exclusively on these qualitative data to answer the question: “Which factors account for cross-country variations in the effectiveness of CRP biomarker test interventions?” Covering nearly one million words worth of transcripts and interview notes from 130 respondents, the extent and granularity of our qualitative data allowed us to carry out a cross-case comparison using thematic analysis [40] and contribute an original perspective to the understanding of contextual factors in clinical interventions. We thereby also contributed to the qualitative research of CRP POCT, which had thus far only involved single-case studies in high-income settings [41, 42].

## Methods

### Study design

The CRP POCT trials were situated in Southeast Asia, which is deemed a global AMR epicentre due to widespread unregulated antibiotic use in humans and animals [43–45]. The qualitative data were collected in a cross-sectional design as part of the clinical trials, involving participating HCWs and patients (see Table 1 for an overview of the clinical trials). The initial intention of this qualitative data collection was to broaden the evidence base and help explain the results of each clinical trial individually [46]. More specifically, the objective of the qualitative data collection in Chiang Rai and Yangon was to contextualise the CRP POCT: “within an existing system of practices at the patient–health system interface” [47]; the Hanoi data were collected to “assess the acceptance of CRP [point-of-care] testing to reduce inappropriate antimicrobial use for self-limiting [acute respiratory infections] among patients [including guardians of child patients] and HCWs in Vietnam” [48]. Although the initial intention of the qualitative data collection was not to carry out a cross-case comparison, the qualitative data sets contained extensive and compatible context-related information (e.g. patients’ conceptions of illness, HCWs’ perceptions of patient expectations) that enabled a secondary analysis across the three trial case studies. We therefore synthesised the qualitative data from the three trials and compiled a large qualitative data set, using inductive thematic analysis (i.e. without predefined themes) to identify factors that accounted for cross-case variation in the effectiveness of the CRP POCT trials [49].

**Table 1:** Clinical trial characteristics

	Case study		
	Chiang Rai (Thailand)	Yangon (Myanmar)	Hanoi (Vietnam)
Study population	Febrile patients	Febrile patients	Patients with acute respiratory infections
Trial sample	1182 Participants (600 adults, 582 children)	1228 Participants (609 adults, 619 children)	2036 Participants (1008 adults, 1028 children)
CRP POCT users^a^	Nurses and public health technical officers^b^	Medical doctors	Medical doctors
Location	Peri-urban Chiang Rai district	Hlaing Tha Yar and Shwe Pyi Thar sub-urbs	Rural and urban Hanoi
Study sites	6 Public primary healthcare centres	3 NGO clinics and 1 public hospital	9 Public primary healthcare centres (urban) and 1 public district hospital (rural)

Source: Authors

*CRP POCT* C-reactive protein point-of-care test, *NGO* non-governmental organisation

^a^“Users” refers here to the healthcare workers who interpreted the test results. The trials involved dedicated study staff to operate the CRP POCT, which would not necessarily be the case in routine settings

^b^For simplicity, we will only refer to “nurses” when considering healthcare workers in Chiang Rai

The target populations for the clinical trial in Hanoi were patients with non-severe acute respiratory infections, which represented a group of particularly high (and potentially ineffectual) antibiotic use in Vietnam [50]. The trials in Chiang Rai and Yangon focused on febrile patients, considering the regional endemicity of malaria alongside the limited range of diagnostic aides to guide treatment for undifferentiated fevers [32]. These target groups are comparable in their clinical presentation and represent the commonest causes of attendance in primary care. The target samples of the clinical trials involved 1200 patients each in Chiang Rai and Yangon and 2000 in Hanoi, each comprising 50% adults and 50% children (4446 trial participants were recruited in total). As part of the consent process, all trial patients were provided with brief information in the local language about the role of antibiotics and the risk of antibiotic resistance.

The clinical trials assessed the effectiveness of a CRP POCT on reducing antibiotic prescriptions at urban, peri-urban, and rural primary-care-level healthcare facilities. As a diagnostic tool, CRP indicates whether a patient is likely to have a bacterial infection, and the CRP POCT was intended to discourage HCWs from prescribing an antibiotic below a certain threshold that indicated the absence of bacterial infections (10 and 20 mg/L in children and adults in Hanoi, respectively; and two intervention arms using 20 and 40 mg/L thresholds in Chiang Rai and Yangon). The CRP POCT was administered as a finger-prick blood test, and a reader device (NycoCard II® reader) reported CRP levels within 5 min (for a more detailed description of the process, see [47]). The participating primary healthcare facilities in Chiang Rai comprised public health centres staffed with nurse practitioners and public health technical officers. In Yangon, the outpatient department of a government hospital and clinics run by a local non-governmental organisation were staffed with medical doctors, and so were the public health centres and the public district hospital in Hanoi. Healthcare staff in all three sites received the CRP POCT results from dedicated study staff, namely study nurses in Chiang Rai and study doctors in Yangon and Hanoi. The HCWs would then use the CRP POCT results to complement their clinical judgement.

### Qualitative data collection

The qualitative sampling strategies and the ensuing samples are summarised in Table 2. In Chiang Rai, all participating HCWs were recruited, whereas the Yangon and Hanoi studies sampled at least one HCW from each participating site depending on their experience with the CRP POCT and their availability. Patient sampling in Hanoi involved a random sample of the patients in the treatment groups (including guardians when the patients were children), recruited successively until data saturation was reached (defined as no new themes arising from two consecutive focus group discussions/semi-structured interviews, which can already occur after 10–12 interviews [51, 52]). In Chiang Rai and Yangon, purposive maximum variation samples were obtained specifically to capture differences across the target population. The variables guiding the patient selection were the randomised allocation to the trial group, antibiotic prescription, sex, age, and education level. To understand the intervention context more comprehensively, the Chiang Rai and Yangon samples also included febrile patients who did not participate in the clinical trial (sampled through patient logs of the healthcare facilities). The resulting qualitative data set involved 130 participants and comprised semi-structured interviews (SSIs, lasting 30–90 min) and focus group discussions (FGDs, lasting 1–2 h).

**Table 2:** Qualitative sample characteristics

	Case study		
	Chiang Rai (Thailand)	Yangon (Myanmar)	Hanoi (Vietnam)
Timing of data collection	August 2016, May 2017	December 2016 –January 2017	June – December 2015
Healthcare worker sample			
Sample size	21 HCWs (16 female/5 male)	12 HCWs (6 female/6 male)	12 HCWs (10 female/2 male)
Sampling strategy	Census (all participating HCWs)	Purposive sample (at least 1 from each site)^a^	Purposive sample (at least 1 from each site)^b^
Semi-structured interviews	21 SSIs	12 SSIs	2 SSIs
Focus group discussions	None	None	1 FGD (10 participants)
Patient sample			
Sample size	37 Patients^c^ (control and treatment; 24 female/13 male, average age 42 years)	21 Patients^c^ (control and treatment; 13 female/8 male, average age 37 years)	27 Patients^c^ (treatment group only; 23 female/4 male, average age 49 years)
Sampling strategy	Purposive sample (maximum variation)^d^	Purposive sample (maximum variation)^d^	Random sample with information saturation^e^
Semi-structured interviews	25 SSIs (incl. 2 interviews with 2 participants)	11 SSIs (incl. 1 interview with 2 participants)	9 SSIs
Focus group discussions	3 FGDs (3 male, 4 female; 3 female guardians)	2 FGDs (4 male, 5 female; mixed adult/guardian)	3 FGDs (5/6/7 participants; male/female/guardian)

Source: Authors

“Guardian” is defined as an interview participant who signed consent for a child participating in the clinical trial, or non-trial respondent who was responsible for care of a child. However, guardians reported on their own health behaviour as well as their children’s.

*HCW* healthcare worker, *SSI* semi-structured interview, *FDG* focus group discussion

^a^Respondents within sites selected on basis of availability

^b^Respondents within sites comprising main study doctors who enrolled more than 80% of the centre’s total sample. At least one such doctor per site would participate in the focus group discussion. In two sites, there were two such doctors; one would participate in the focus group discussion and one each would participate in a semi-structured interview

^c^Including patients and guardians of patients who were children

^d^Maximum variation across the following variables: patients’ study groups (pre-intervention/control/treatment group), antibiotic prescription (yes/no), sex (male/female), age (guardian of a child below 18 years/18–49/50+), education (below/above primary education)

^e^Saturation criterion: no new themes arose from two consecutive focus group discussions/semi-structured interviews

The topics of the data collection instruments were based on the literature around frontline HCWs, treatment seeking and antibiotic use in LMICs, and rapid diagnostic testing for malaria [53–55]. SSI and FGD guides covered similar topics, which are summarised in Table 3 (the complete interview guides are presented in [47, 48]). The FGDs focussed on triangulating insights from the SSIs and therefore did not explore the topics in the same level of depth. All interviews and discussions were conducted in the local languages (Burmese, [Northern] Thai, Vietnamese) and yielded approximately 98 h of audio-recorded material. The audio records were transcribed and translated by the study team members who conducted the interviews. The transcripts were complemented with written notes describing for instance the interview setting and non-verbal communication. The resulting 977,000 words of written material formed the basis of our qualitative data analysis.

**Table 3:** Qualitative data collection topics and example questions

Patients in Chiang Rai and Yangon		Patients in Hanoi	
Data collection topics	Example questions	Data collection topics	Example questions
Medicine use and treatment-seeking behaviour	“You recently visited the health centre because of a fever. What was the process of getting treatment? Please be as specific as possible, step by step.”	Acute respiratory infections (ARIs) and treatment-seeking behaviour	“What is your understanding about the causes of ARI and its natural history?”, “Why did you choose to visit the clinic on this occasion?”
Decision-making about medicines	“When would you use medicines for an illness? When not?”	Perception of CRP testing	“Does the test need to be improved? If yes, how?”
Demand-side preferences, local notions and myths about medicine	“What is the best treatment for fever?”	Impact on antimicrobial use	“What do you expect from seeing the doctor with ARI?”, “Did you seek for subsequent antimicrobials if your doctor did not give you antimicrobial?”
Health provider landscape and preferences from patient perspective	“Can you tell me which health providers are available to you, and which of them you would visit for treatment?”	Impact on consultation	“What other information would you need to help you fully trust the test and trust the doctor’s opinion that you do not need antimicrobials?”
Experiences in public healthcare	“For your visit at the health centre, can you please tell me: How did you feel if you did not receive the medication you expected?”	Recommendations	“In your opinion, should a CRP test be done as a part of routine diagnosis for ARI patients in primary care settings?”
CRP POCT experiences	“Do you feel that you were treated differently than usual because of the test?”		
Healthcare workers in Chiang Rai and Yangon		Healthcare workers in Hanoi	
Data collection topics	Example questions	Data collection topics	Example questions
Workload, freedom and constraint in work	“What are your roles and responsibilities in your work”	Perception of CRP testing	“What do you like / dislike about the test?”
Scope of outpatient work	“How many outpatients do you deal with on a normal day”	Impact on antimicrobial prescription	“How did the test support your treatment decision?”, “What do you think your patients are expecting from seeing a doctor? (Drugs / Antimicrobials / Advice / Reassurance / Diagnosis / Others)”
The system context of CRP POCT	“Are any tests being carried out (e.g. by yourself) to diagnose [common outpatient complaints]?”	Impact on consultation	“Did you use the CRP result to discuss with patients about your treatment decision?”
Antibiotics marketing	“Do drug company representatives promote the use of certain medicines in your health centre?”	Recommendations	“In your opinion, should a CRP test be introduced in routine practice of your setting? Why / Why not?”
Extent of patient demand, dynamics in patient–HCW interaction	“Do patients demand certain drugs or treatments?”		
Antibiotics prescription practice	“For what conditions do you prescribe antibiotics?”		
Risk reduction through antibiotics	“Can antibiotics be a way to protect you from patient demands, ineffective treatment, or problems in diagnosing an illness?”		
(Measures to limit) over-prescription	“If you had to reduce antibiotics prescriptions, what would you consider the most effective way?”		

Source: Haenssgen et al*.* [47], Do [48]

Healthcare worker (HCW) interviews in Chiang Rai and Yangon initially included vignettes to explore understanding of best practices, which were dropped due to time constraints

*CRP* C-reactive protein, *CRP POCT* C-reactive protein point-of-care test

### Qualitative data analysis

Considering the scarcity of knowledge on contextual factors and their “subjectivity” [3], we chose an inductive thematic analysis approach for this cross-case comparison. This means that we developed themes from the narratives of patients and HCWs who were involved in the trials (although different from a “grounded theory approach,” this is sometimes referred to as “grounding” themes in the qualitative data [56]; the inductive approach is opposed to a deductive approach that would explore themes derived from theory or the literature [49]).

The trial outcomes that we considered were (1) patient adherence and (2) HCW (or “clinical user”) adherence to the biomarker test results and (3) patient exclusion, to gauge the potential “impact” of CRP point-of-care testing on the population level (e.g. even if user and patient adherence were high, the trial impact may be diminished if the CRP POCT only reaches a small fraction of the population). Following the process description for qualitative cross-case comparisons by Bazeley [40], the analysis took place in 3 stages: first, description of each individual case study; second, identification of similarities and differences of each case in relation to the trial outcomes through pairwise comparisons; and third, interpretation of key variables influencing the trial outcomes in the three case studies.

In stage 1, all textual material was read and coded using Nvivo 11 [57]. Codes were assigned in relation to any of the 3 outcomes, starting with the SSI transcripts as our primary data source, which we triangulated with FGD transcripts (interview notes provided an overview and contextualising information). The triangulation through FGD transcripts involved checks of completeness and representation of statements from the SSIs, whereby we analysed participants’ contributions individually in the context of the dynamic evolution of the discussion (see [58]; this corresponds to participant-based group analysis, as opposed to whole-group analysis [59]). In order to ensure consistency of the coding frame across the three case studies, this stage involved two coding rounds of all textual material and was conducted in a single-coder approach by the lead social scientist (MJH; ambiguities in meaning in the translated transcripts were clarified during this stage through the local study team members NC, NTTD, YKZ).

In stage 2, each individual case study was compared pairwise against the remaining two cases, the similarities and differences of which were tabulated in a cross-case comparative matrix. After a first round of comparative coding by the lead social scientist, the matrix was validated and amended by the local study team members who were involved in the qualitative data collection (NC, NTTD, YKZ), and by the team members who coordinated the clinical trials across the three countries (TA, NTTD). This validation process involved the critical interrogation and challenge of the identified themes (e.g. in light of the original-language transcripts) and the consideration of alternative or omitted themes. This joint deliberation entailed a further round of analysis of all textual material relating to challenged or omitted themes.

In stage 3, the study team derived jointly the key themes of the analysis, namely contextual domains, outcomes, and the pathways linking them. In light of space constraints, we focussed our presentation of the results on the key themes and the cross-case comparative matrix; an in-depth description of each individual case is provided in Additional file 1. We further refrained in this paper from linking quantitative outcome measures to the qualitative themes, considering the breadth of the latter (in statistical terms, we would have fewer observations than variables to specify the exact relationship).

## Results

**Fig. 1:**
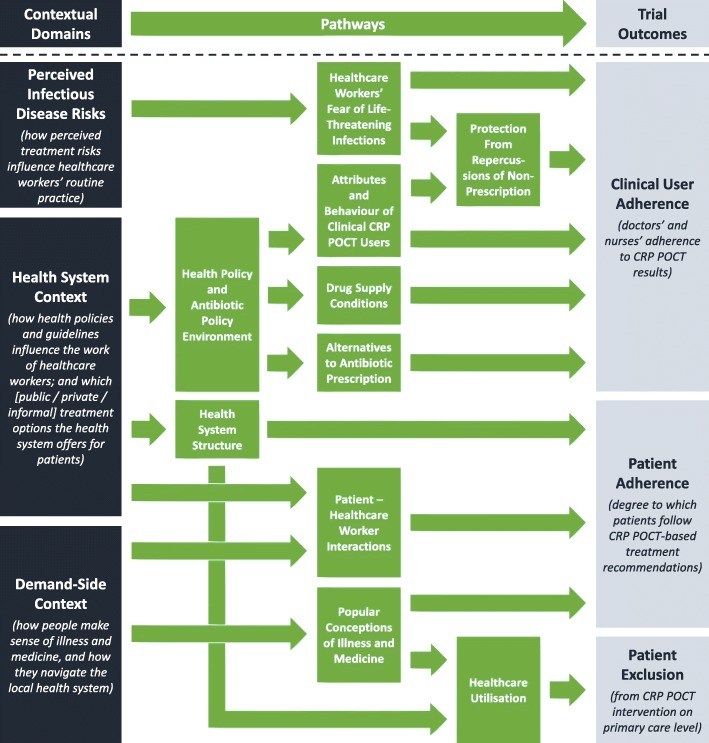
Contextual factors influencing C-reactive protein point-of-care test (CRP POCT). Source: Authors, derived from qualitative analysis. “Health systems” here comprise all formal and informal actors involved in promoting, maintaining, or restoring health according to the World Health Organization [91], which can include for example medicine-selling grocery stores alongside public and private hospitals

Figure 1 summarises the key themes of our qualitative analysis and illustrates the pathways (depicted in green) through which the CRP POCT trial outcomes (dark blue) were affected by three inter-related domains of contextual factors (light blue). The first domain was perceived infectious disease risks, defined as treatment risks stemming from the disease environment as HCWs evaluate them in their routine practice (rather than “objective” measures of disease burden in the local area). Second, the health system context comprised health policies and guidelines that governed the work of healthcare workers, and the structure of the health system. Third, the demand-side context related to local healthcare-seeking behaviours and popular conceptions of illness and medicine, and the resulting utilisation of the health system among patients. We exemplified the concrete manifestations of these elements across our three case studies in Table 4 and described the main elements in detail in the remainder of this section, structured according to the three contextual domains.

**Table 4:**
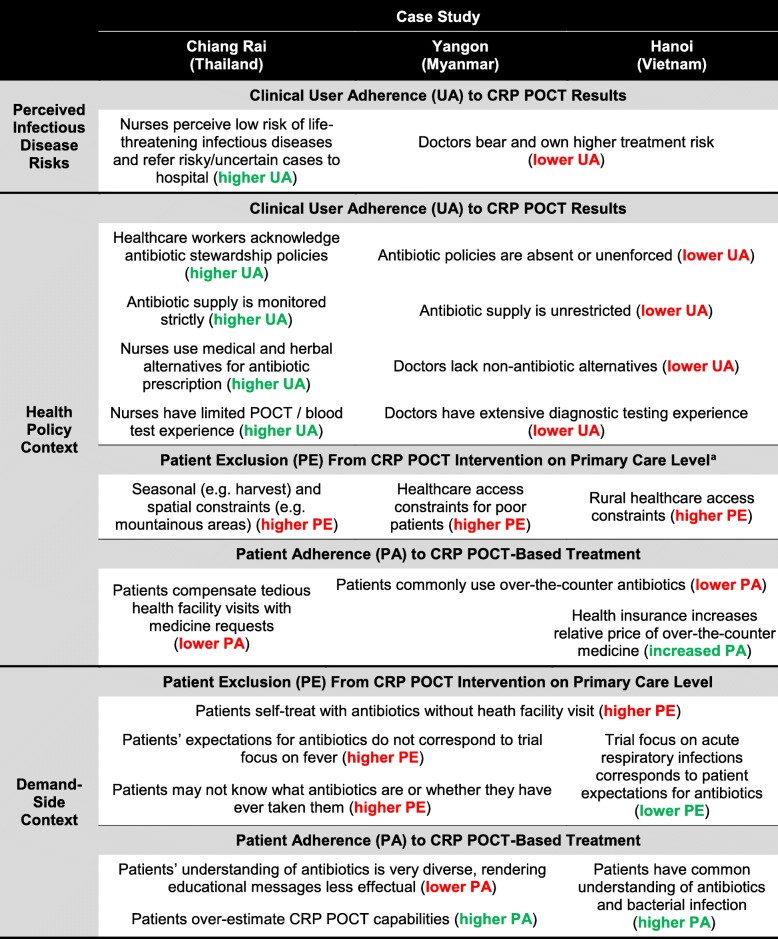
Summary of contextual impact on outcomes of clinical trials

Source: Authors, derived from qualitative analysis

Higher patient exclusion (PE) and lower user/patient adherence (UA, PA) correspond to a negative impact of contextual factors on trial outcomes and are indicated in red; lower exclusion and higher adherence are indicated in green. References to “patients” do not imply uniform responses of the target group

^a^Items in this category relate to the type of patient being excluded from the intervention

### Perceived infectious disease risks

The first contextual influence involved HCWs’ perceptions and fears of potentially life-threatening infections and the extent to which they owned and managed the associated treatment risks (e.g. through referral). A doctor in Hanoi suggested for instance that antibiotic prescription decisions related partly to individual risks that the clinical users (i.e. HCWs) of CRP POCT faced in their treatment:

“If we prescribe antibiotics, we would not be blamed for any problem the patients might have. If we don’t prescribe antibiotics, the patients might get worse. In this case, we would not be able to explain to their relatives. And they would not accept our explanation.” (Doctor, Hanoi, FGD)

Perceptions of risks may thereby correspond only partially to the actual epidemiological environment. For example, a survey in Vietnam by Minh [60] detected very low rates of pneumonia of 1.2% among 563 outpatients with acute respiratory infections visiting a paediatric tertiary referral hospital [60]. Yet, doctors in Hanoi repeatedly expressed the need to protect themselves from potentially under-treating such infectious diseases and “co-infections of both viral and bacterial infections” by over-prescribing (Doctor, Hanoi, SSI).

We observed similar tendencies in the Yangon clinics, which catered especially to patients with tuberculosis (TB) and patients with HIV and which were situated among squatter populations with poor hygiene and environmental conditions [61, 62]. The participating doctors would thus describe common risks of co-infection and that: “If [the CRP test result is] low and [the patient’s] condition is bad, and there is bacterial infection, what we fear most in the bacterial infection is the pneumonia. So for that we would give [antibiotics] even if the CRP is low” (Doctor, Yangon, SSI). Considering the possibility of co-infection, antibiotics were also often prescribed as prophylactic treatment especially in patients whom the doctors considered to be high-risk groups (e.g. children, malnourished patients, or those from lower socio-economic backgrounds: “some [patients] are very weak, so then I give antibiotics, but for patients with asthma or heart failure by birth, or children, I give antibiotics even if they are not weak, because they are more prone to infection” Doctor, Yangon, SSI).

In contrast to the experiences from Yangon and Hanoi, respondents in Chiang Rai would only occasionally express a need for prophylactic prescriptions, considering the different patient profiles resulting for example from higher average wealth and access to improved sanitation facilities (e.g. access to improved sanitation in Thailand was 93% in 2015, compared to 78% in Vietnam and 80% in Myanmar [63]; see Additional file 1 for details). However, when for example, pneumonia was suspected, one nurse explained that: “mostly we’d have to refer and a medical doctor will take care of it because that’s something quite serious” (Nurse, Chiang Rai, SSI). Referral mechanisms therefore mitigated the remaining risks for the nurses when patients had low CRP but suspected pneumonia, which underlined the role of the health policy environment as a further determinant of the risks that HCWs perceived when not prescribing an antibiotic.

In summary, perceived infectious disease risks undermined user adherence with CRP POCT insofar as they created a fragile balance between clinical judgment and the fear of missing a bacterial infection. Perceived infectious disease risks when refusing patients antibiotics on the basis of a negative CRP POCT could include social pressure and adverse patient outcomes. Such situations appeared more pronounced in Yangon and Hanoi: doctors more commonly articulated fears of undertreating potentially life-threatening infections and they owned treatment risks to a greater extent than the nurses in Chiang Rai.

### Health system context

#### Policy environment

The health policy environment related to three important themes in our data: (1) the supply environment of antibiotics, (2) the range of available alternatives to antibiotic treatment, and (3) the characteristics of HCWs as users of the CRP POCT.

First, the supply environment contributed to the liberty with which HCWs could prescribe antibiotics. With the 2007 Antibiotic Smart Use campaign and the 2017–2021 National Strategic Plan on Antimicrobial Resistance, Chiang Rai experienced a higher-level drive towards better stewardship alongside local initiatives like stricter monitoring of primary-care-level antibiotic prescriptions [64–67]. A health centre director described the situation as follows: “the policies from the ministry […] would focus [increasingly] on antibiotics. […] It’s the kind of work of which if we don’t reach the goal, our expenses or income and things like that from that particular performance will decrease” (Nurse, Chiang Rai, SSI). In this strict supply environment, the CRP POCT emerged as a complementary tool to help meet the policy requirement of lower antibiotic prescription.

In contrast, health policy in Myanmar and Vietnam had hitherto focused on expanding the availability of antibiotics, while existing AMR-related policies in Vietnam had remained largely unenforced (e.g. the 2005 Drug Law; [45, 50, 68–70]). One manifestation of the comparatively lax regulation was that HCWs experienced no supply restrictions when prescribing antibiotics (notwithstanding the limited spectrum of available antibiotics). A doctor in a non-governmental organization (NGO) clinic in Yangon described for instance that antibiotics were well-stocked compared to other medicines: “We may run out of stock for other medicines but not the antibiotics. Because I think that according to seasonal needs during these months it’s quite high and we have a lot more consultation” (HCW, Yangon, SSI). Such availability of antibiotics was even more pronounced in Hanoi, where some respondents experienced a supply glut of antibiotics to an extent that would render the CRP POCT almost superfluous:

“100% of patients have been provided with antibiotics as here are a lot of antimicrobials in stock that need to be dispensed. Depending on CRP results, I tell my patients to use the antimicrobial immediately or keep for another illness episode.” (Doctor, Hanoi, FGD)

In other words, adherence appeared to drop in conditions of lax regulation and abundant supply.

Second, the presence and promotion of alternatives to antibiotic prescription influenced adherence as well. When asked whether she would prescribe antibiotics to insistent patients, a nurse in Chiang Rai for example explained: “Sometimes I would change to herbal medicines instead because here we have herbal medicines. Instead of antibiotic, I can give them Fah Talai Jone [ฟ้าทะลายโจร] to avoid their antibiotic use, I can use that technique” (Nurse, Chiang Rai, SSI). In Chiang Rai, such alternatives were promoted by antibiotic stewardship initiatives and complemented both the strict regulatory environment and the CRP POCT intervention (another common technique was to defer prescription [47]). The opposite situation materialised in Yangon. The participating NGO clinics (specialising in TB and sexually transmitted infections) would stock only a narrow range of general medicine like cough suppressants. This meant that doctors had to rely on antibiotics for want of more appropriate choices: “If we can get other suppressants, other supported treatment, then we wouldn’t use antibiotics when we hear crepitations” (Doctor, Yangon, SSI). The limited range of non-antibiotic medicine for general patients therefore undermined doctors’ ability to adhere to a negative CRP POCT result.

Third, the policy environment also shaped the characteristics of HCWs, for example their awareness of antibiotic resistance. It was common in all three contexts for HCWs to ignore antibiotic resistance as a local problem relevant to their routine practice:

“I don’t think [antibiotic over-prescription] is a problem in health centres. Because you need to prescribe it anyway, it’s a principle. If you don’t, the patients cannot get better.” (Nurse, Chiang Rai, SSI)

“It is not the problem of my clinic. We do not have the pressure of prescribing antibiotics.” (Doctor, Hanoi, FGD)

“Doctors mainly have limitations [i.e. guidelines when using antibiotics], but I think that the drug stores are out of control. Doctors have their ethics so …” (Doctor, Yangon, SSI)

However, the comparatively active policy environment in Chiang Rai meant that the nurses had been widely exposed to the problem through national policies (“they want us to focus on [antibiotic] ‘Smart Use’ [a campaign to raise awareness and reduce antibiotic use];” (Nurse, Chiang Rai, SSI)), operational guidelines, and the media (“a lot of us [nurses] began to use social [media] now so that increases the knowledge for us;” (Nurse, Chiang Rai, SSI)), while also recognising a greater degree of public awareness (“the patients learn from the media [i.e. TV], as well” (Nurse, Chiang Rai, SSI)). The implementation of the CRP POCT in this environment resonated with the existing degree of antibiotic stewardship. We could not discern such a link in the weaker AMR policy environments of Yangon and Hanoi. A doctor in Yangon indicated for instance that, “Oh, we don’t have it here [i.e. initiatives to reduce antibiotic use]” (Doctor, Yangon, SSI).

A final example of health policy context was its influence on primary-care-level antibiotic prescribers’ prior experience with point-of-care and laboratory tests. Considering that only a few diagnostic technologies were available at the primary care level in Chiang Rai (e.g. finger-prick blood glucose testing [71]), the introduction of a novel point-of-care test was often received favourably by the participating nurses. For example, a nurse described that: “I check on the patients and they would feel, like, like, ‘Our hospital [i.e. health centre] is modern,’ you know? […] It’s like it’s upgraded our class to something higher, and we seem better” (Nurse, Chiang Rai, SSI). The technological enthusiasm was not echoed in Yangon and Hanoi, where the doctors were familiar with a range of diagnostic testing technologies and their hospital and specialised clinic environments offered a variety of testing facilities and routine blood tests (“frankly speaking, we can get X-ray and do something more informative;” (Doctor, Yangon, SSI)). Nuanced and conservative attitudes towards the intervention based on broader experiences with diagnostic technologies therefore suggested a lower degree of reliance on and adherence to the CRP POCT in Yangon and Hanoi.

In summary, our interviews indicated a strong link between the health policy environment and HCWs’ adherence to the CRP POCT intervention. The policy environment shaped the antibiotic supply environment and the monitoring thereof, the availability of alternatives to antibiotic treatment, and the characteristics of the primary-care-level users of CRP POCT. In Chiang Rai, this created complementary conditions for the intervention and reinforced nurses’ trust in and adherence to CRP POCT. The opposite was the case in Hanoi and Yangon, where unrestricted antibiotic supply together with a lax regulatory environment, limited concerns about antibiotic resistance in HCWs’ routine practice, and experience with a wide range of diagnostic technologies appeared to undermine adherence.

#### Health system structure

The primary healthcare centres hosting the Chiang Rai trial were free of charge (except for unregistered minorities) and commonly accessed by poorer segments of the population, provided that these facilities were neither overcrowded or out of reach [47, 72]. The Yangon study clinics provided free healthcare as well, but were located in poor sub-urban slums with widespread unregulated access to antibiotics and unlabelled medicine sets (so-called “drug cocktails” [61, 62, 73]). In Hanoi, the participating clinics were commonly accessed by the poor and people with health insurance, but the first and cheaper step during an illness was typically self-medication [48, 68]. Our qualitative analysis suggested that such health system configurations influenced patients’ adherence to CRP-POCT-based treatment, but they also determined the population groups who were excluded from routine access to healthcare and thus from the intervention.

Local health system structures shaped the range of available healthcare choices for patients and thereby influenced their adherence to CRP-POCT-based treatment. For instance, patients who incurred a time-consuming and costly visit to a primary healthcare care facility often articulated an expectation to receive some form of medication to not leave the health facility empty-handed. This was particularly pronounced in Chiang Rai, where patients’ responses often reflected explicit expectations for medicines and a sense of entitlement (“I take time to go to the doctor, if they don’t give [medicines] I’d be sad” (patient, Chiang Rai, SSI)). Similar expectations were common in Hanoi (“Some patients requested for more drugs so that they would not have to come back to the clinics at next time of being sick” (Doctor, Hanoi, FGD)), also because health insurance coverage appeared to stimulate medicine expectations (“I go to the clinics because my house is very close to this clinic and medicines are covered by health insurance. So I don’t go to drug store because I have to pay for medicines” (patient, Hanoi, SSI)). The patients interviewed in Yangon had generic expectations of medicine (rather than antibiotics in particular: “The unofficial unprescribed medicines are not helpful so we come in hopes that medicines from here would cure us” (patient, Yangon, SSI)), but also seemed to access informal and private sources of medicine commonly prior to the clinic/hospital visit (“Before [coming to this clinic], I would just take the mixed medicines [“drug cocktails”], I didn’t go to the clinic” (Patient, Yangon, SSI)).

The health system structure also entailed target group heterogeneity in terms of exclusion from routine primary healthcare access, which shaped the potential population-level impact of the intervention. For example, a doctor in Hanoi described that: “Patients in rural areas which are far from the hospital could not come back for re-consultation, so [they] treated themselves with antibiotics at home” (Doctor, Yangon, SSI). In Chiang Rai, villagers living in mountainous areas would cite healthcare access constraints like: “If we don’t have money, we would borrow and go buy [medicines] near our house [rather than going to the hospital]” (patient, Chiang Rai, SSI). Also, seasonal constraints were mentioned, with the workload around the rice harvest meaning that: “most people would come [to the health centre] after they’re done harvesting” (Nurse, Chiang Rai). Access to formal healthcare (and thus to the CRP POCT intervention) would therefore be limited, especially for poor people in rural and mountainous areas and during harvest the season.

In short, the structure of the formal and informal health system determined whether other healthcare providers like pharmacies, private clinics, or even local grocery stores could absorb patients’ demands for antibiotics. Yet, healthcare access constraints like poverty and remoteness led to the exclusion of parts of the relevant target groups in all three case studies.

### Demand-side factors

The third and final domain of contextual factors related to the demand side of healthcare services, influencing patients’ adherence to the CRP POCT and exclusion from the intervention. Patient adherence was affected when patients challenged the authority and decisions of HCWs. This was especially pronounced in Chiang Rai, where nurses rather than doctors were involved in the clinical intervention (e.g. “they’re not doctors here [at the health centre], they’re nurses” (patient, Chiang Rai, SSI); versus “I have gone to hospital whenever I am ill. I trust in doctors” (patient, Hanoi, FGD)). In addition, in both Yangon and Chiang Rai (for which we had more comprehensive qualitative data), patients with less formal education, from lower socio-economic strata, or with ethnic minority backgrounds would appear less assertive and more compliant with HCWs’ treatment decisions, stating for instance that: “I don’t have any knowledge, so I’d take anything. I’d take whatever they advise” (patient, Chiang Rai, SSI). Healthcare workers echoed this observation and described these patients as being “easy to talk to” (Nurse, Chiang Rai, SSI) and that they “don’t understand about medicines, so they do accept the treatment we give” (Doctor, Yangon, SSI). Based on these examples, we hypothesise that patient adherence is higher in settings where the distance in power between HCWs and patients is larger.

Adherence to the CRP POCT results could be further undermined if patients’ conceptions of illness and medicine were at odds with the implicit logic of the intervention (*viz*. a conceptual distinction between bacterial and non-bacterial causes of illness to guide antibiotic prescription). With few exceptions, SSI and FGD respondents in Hanoi articulated a working concept of “bacteria” and “viruses” as disease-causing agents. This conception was less prevalent among our respondents in Chiang Rai and Yangon, who would often link illness to an “inflammation” of the body (Chiang Rai) or to an infection with generic “germs” (Yangon). Respondents in these two sites also had a wider range of notions of antibiotics, which would include “anti-inflammatory medicine” (Chiang Rai), “germ killers” (Chiang Rai, Yangon), or “pesticides” (Yangon), and some patients especially in Yangon did not: “quite understand what germ killers [i.e. antibiotics] are for” (patient, Yangon, SSI). As local conceptions of illness and medicine in Chiang Rai and Yangon more often contradicted the biomedical logic of the CRP POCT, the information to explain the test might have been less effective than in Hanoi. At the same time, we observed a common pattern in Chiang Rai and Yangon that patients misinterpreted and over-estimated the capabilities of the CRP POCT as a comprehensive blood test (e.g. the finger-prick test indicating: “[…] whether this disease is good or bad, or if it’s very serious or not. And we get to know what disease it is […]” (patient, Yangon, SSI)). Ironically, this discrepancy appeared to increase rather than undermine patient adherence (see [47, 73] for more discussion on this point).

Local conceptions of illness and medicine also affected exclusion from the CRP POCT: first, local approaches to self-treatment with antibiotics were common in all three sites, and they could potentially involve strategies as elaborate as described by a patient in Hanoi:

“Sometimes I give [my daughter] ampi [ampicillin], small capsule. After replacing it by cefexim, I found [the treatment] better. Since then, I often treat her with cefixim at home, normally for 3-5 days. If she doesn’t have fever, I will treat her at home or buy medicines from [the] drug store.” (Patient, Hanoi, SSI)

While self-treatment with antibiotics was shaped partly by local conceptions of illnesses and their corresponding remedies, it was also an expression of barriers to accessing healthcare: “If [the patients] are really a hill tribe member, I don’t see them participate [in the trial], I don’t think. Because it’s hard for them to come down [from the mountain], something like that. It’s hard, it’s inconvenient” (Nurse, Chiang Rai, SSI)

Second, a mismatch emerged in Chiang Rai and Yangon between patients’ expectations about antibiotics and the focal condition of the test: neither patients nor HCWs would commonly demand antibiotic treatment for a fever, unless accompanied by other symptoms (see [71] for an analysis of administrative primary-care-level data from Chiang Rai):

“Anti... anti-inflammatory [i.e. antibiotic]; if they have a fever only—fever or cold—I wouldn’t prescribe [an antibiotic]” (Nurse, Chiang Rai, SSI).

Question (Q): “Right. And when you have a fever, do you normally take anti-inflammatory [i.e. antibiotic]?”

Response (R): “For just fever, no, only Para.”

Q: “There has to be a sore throat.”

R: “Yes, if there’s an irritation, I’d take it right away.” (patient, Chiang Rai, SSI).

“Here they don’t ask for germ killers [i.e. antibiotics]. Because people that come here don’t have much knowledge, they might not even know that what they are taking are germ killers.” (Doctor, Yangon, SSI).

“I don’t take medicine [for a fever]. I usually have a sponge bath, if I have doubts [that I have fever], I take a sponge bath. I don’t usually take medicine.” (patient, Yangon, SSI).

Owing to the incongruency between fever and antibiotic demand, a doctor in Yangon reflected that: “I don’t think that it [i.e. CRP POCT] can change much the amount of antibiotics [on the clinic level] based on whether or not to give antibiotics to those 5 or 10 people [out of 200 patients/day]” (Doctor, Yangon, SSI).

In summary, a smaller distance in power between HCWs and patients and discrepancies between the intervention logic and the local conceptions of the target population appeared to undermine patients’ adherence to the CRP POCT results. In addition, incongruencies between local forms of antibiotic use and the disease/healthcare provider focus of the CRP POCT intervention could diminish the potential overall impact at the population level.

## Discussion

### Summary

The objective of this paper was to contribute to the understanding of how contextual factors influence the implementation and operation of medical interventions. We compared three case studies of CRP POCT trials involving qualitative research with 130 healthcare workers and patients across Yangon (Myanmar), Chiang Rai (Thailand), and Hanoi (Vietnam). The qualitative cross-case comparison demonstrated how perceived infectious disease risks, health system factors, and the demand-side context systematically influenced clinical trial outcomes (adherence of HCWs and patients to the test results, and target group exclusion from the clinical trial). From a HCW perspective, less pronounced fears of undertreating infectious diseases by withholding antibiotics, stricter prescription monitoring, and the promotion of alternatives to antibiotic treatment appeared to reinforce adherence to the CRP POCT in Chiang Rai. The opposite was the case in Yangon and Hanoi, where absent or unenforced AMR policies appeared to undermine compliance. Patient adherence to CRP-POCT-based treatment was affected positively in Yangon and Chiang Rai, where patients tended to interpret the CRP POCT as a comprehensive blood test and therefore had a higher degree of trust in the intervention. A final example was the disease focus of the trial, which did not correspond closely with expectations about antibiotic treatment among doctors and patients in Chiang Rai and Yangon. This mismatch may have entailed a relatively higher degree of exclusion of antibiotic users among the target population compared to Hanoi.

### Implications

The documented variation in contextual factors demonstrated how similar clinical trials operated differently across countries and different parts of their target populations. The operational variations could influence the interpretation and generalisability of trial findings. For example, a CRP POCT trial implemented without a complementary policy environment or out of sync with local expectations of antibiotic use may yield less significant findings than otherwise, which could hinder the pursuit of further research in single-site trials unless the source of the contextual impact is clear. Likewise, trial results could appear positive yet emerge as unsustainable in routine practice if healthcare workers reverted to their accustomed behaviours during the workload-intensive monsoon season.

While these implications were specific to the CRP POCT trials, we could also distil more general lessons for AMR-related interventions and for other clinical trials more broadly. For AMR-related interventions, health system factors appeared to be of fundamental importance and echoed related social sciences research on malaria rapid diagnostic testing in low-income and middle-income countries. The literature has highlighted such factors as the drug supply environment, healthcare workers’ prior experience with diagnostic testing, or the availability of alternative treatment options in case of negative test results [14, 74, 75]. If AMR interventions failed to appreciate the local context (e.g. the nature of the drug procurement and monitoring systems, existing informal practices of healthcare staff), then they risk duplicating other solutions, competing with existing practices, or producing unintended consequences that potentially undermine the purpose or sustainability of the intervention. The parallels between our study and the literature on malaria therefore underscore that interventions to manage and reduce antibiotic prescriptions need to respond and adapt to the local health system context [76].

The same logic holds for the demand-side context of AMR-related interventions. We highlighted the role of local conceptions and how the resulting interpretations of the CRP POCT affected patient adherence with the intervention. The theme of language and the translation of scientific into popular knowledge is not new and had been raised as an issue in the field of AMR-related behaviour change communication as well [77, 78]. Language and popular conceptions of illness thereby emerged as an important pointer for contradictions between implicit assumptions of the AMR intervention and local realities, which our comparative case study confirmed.

These points may apply to other types of clinical trials, although we cannot sustain this hypothesis without further research. Nevertheless, our work did relate to fundamental contextual impact on clinical trials in diverse settings. One problem is the potential disjunction between interventions’ assumptions and local realities. This was for instance reported in the case of directly observed therapy (DOT) as the World Health Organization’s recommended strategy for TB treatment [79, 80], which faced numerous ethical and logistical challenges like insufficient healthcare resources and insensibility to the socio-economic constraints of patients [81–83]. Our case study was a further example of such tension between internationally recommended guidelines for disease management and local health systems - manifest in the often unrealistic delayed strategy of antibiotic prescription for uncertain diagnoses [84].

Another element of common relevance for clinical trials was target group heterogeneity. Our cross-case comparison illustrated how socio-economically disadvantaged parts of the relevant target population behaved systematically differently from majority groups and struggled with participation in the trial. Such situations may not be uncommon in other low-income and middle-income contexts, where poverty often renders health expenditure catastrophic [85]. Our case study therefore related to methodological arguments in the clinical trials literature according to which the treatment effect of a trial may be shaped by population heterogeneity and selection biases, and average effects may not correspond to effects of the intervention on sub-populations [86–88]. We argue that the characteristics and behaviours of the target group are important demand-side factors that require complementary qualitative evidence to help design and interpret clinical trials within the local context.

### Limitations

The main limitations of our research related to the slightly different implementation of the clinical trials across the three countries, to the varying depth of the qualitative data across our field sites, and to the inductive thematic analysis of our case studies. First, the trial implementation differed for example in terms of the staff involved. Nurses worked at the primary care units and dedicated study nurses supported the clinical trial in Chiang Rai, while doctors and dedicated study doctors participated in the trials in Yangon and Hanoi. In Chiang Rai, the study nurses voluntarily fulfilled other functions that helped ease the workload at the health centres. This unintended trial design effect (which we did not observe in the other sites) may have increased nurses’ compliance with the trial during peak times, *ceteris paribus*. The trial specifications therefore evidently contributed to part of the variation across our case studies. While this limited the comparability of the trials to a certain degree, it was also partly a result of necessary local adaptation, it highlighted the interactions between trial specifications and context [10], and the broadly comparable interventions enabled us to isolate contextual factors nonetheless.

Second, our themes potentially over-emphasised the cases of Chiang Rai and Yangon, where more extensive qualitative data were collected. However, all data sets involved oral accounts from a wide array of healthcare workers and patients, and all data were analysed by local native speakers with knowledge of the CRP POCT trials to provide as much depth to the interpretation as possible. Because the emerging themes applied across all three case studies, we were confident that our analysis uncovered relevant contextual factors.

Third, inductive thematic analysis was chosen to identify unforeseen and locally specific factors on the basis of our informants’ narrative accounts. This meant that we were unlikely to produce an exhaustive list of all contextual factors at work in the three clinical trial case studies - especially if our respondents did not allude to them directly or indirectly, or if they applied equally across Myanmar, Thailand, and Vietnam. For example, while regional and national trade policy regimes may have influenced trial operations through the demand and supply of antibiotics [89, 90], they did not emerge as a theme in the narratives of patients and healthcare staff. Further mixed-method research is therefore necessary to establish a geographically and conceptually comprehensive knowledge base of contextual factors affecting clinical trial outcomes.

## Conclusion

Through a qualitative cross-case comparison involving the narrative accounts of 130 respondents and nearly one million words of transcribed material, we studied the influence of contextual factors on the effectiveness of clinical trials across three Southeast Asian countries. These trials of diagnostic biomarker tests were situated against the backdrop of antimicrobial resistance as a global health priority, wherein our focus on low-income and middle-income countries in Southeast Asia offered direct insights into the realities of interventions in a global AMR epicentre. We thus contributed essential knowledge in global health and in an under-researched area of clinical trials.

We identified three major domains of contextual impact on clinical trials - perceived infectious disease risks, health system factors, and the demand-side context. Yet, rather than providing a definitive list of contextual factors, our study should be understood as underscoring the importance of contextual variation in determining the effectiveness of clinical trials and the meaning of their findings. Further research should investigate the range and magnitude of contextual effects on trial outcomes through meta-analyses of large sets of clinical trials, and identify contextual variables that should be included as covariates. For this to be possible, clinical trials should collect further contextual information including their disease, health system, and demand-side environment - qualitatively and quantitatively. Ultimately, this could help to develop a “toolbox” for clinical trial designers to appraise the viability of a trial in light of its local context, and to capture the most important contextual factors during trial operation in order to interpret and situate their findings.

## Additional file

Additional file 1: Case study overview. (DOCX 114 kb)

## Abbreviations

AMR: Antimicrobial resistance
CRP POCT: C-reactive protein point-of-care test
DOT: Directly observed therapy
GDP: Gross domestic product
HCW: Healthcare worker
HIV: Human immunodeficiency virus
LMIC: Low-income and middle-income country
NGO: Non-governmental organisation
PA: Patient adherence
PE: Patient exclusion
PPP: Purchasing power parity
SSI: Semi-structured interview
TB: Tuberculosis
UA: User adherence
WHO: World Health Organization

## References

[1] Nabi J, Bump JB. Implementing healthcare interventions: context is key. In: Demaio S, editor. PLOS Global Health; 2018. https://blogs.plos.org/globalhealth/2018/04/implementing-healthcare-interventionscontext-is-key/. Accessed 4 Oct 2018.

[2] Pfadenhauer LM, Mozygemba K, Gerhardus A, Hofmann B, Booth A, Lysdahl KB, Tummers M, Burns J, Rehfuess EA (2015). Context and implementation: a concept analysis towards conceptual maturity. Z Evid Fortbild Qual Gesundhwes.

[3] McCormack B, Kitson A, Harvey G, Rycroft-Malone J, Titchen A, Seers K (2002). Getting evidence into practice: the meaning of ‘context’. J Adv Nurs.

[4] Damschroder LJ, Aron DC, Keith RE, Kirsh SR, Alexander JA, Lowery JC (2009). Fostering implementation of health services research findings into practice: a consolidated framework for advancing implementation science. Implement Sci.

[5] Hoddinott P, Britten J, Pill R (2010). Why do interventions work in some places and not others: a breastfeeding support group trial. Soc Sci Med.

[6] Reynolds J, DiLiberto D, Mangham-Jefferies L, Ansah EK, Lal S, Mbakilwa H, Bruxvoort K, Webster J, Vestergaard LS, Yeung S (2014). The practice of ‘doing’ evaluation: lessons learned from nine complex intervention trials in action. Implement Sci.

[7] Fuller D, Potvin L (2012). Context by treatment interactions as the primary object of study in cluster randomized controlled trials of population health interventions. Int J Public Health.

[8] Kent DM, Kitsios G (2009). Against pragmatism: on efficacy, effectiveness and the real world. Trials.

[9] Candy B, King M, Jones L, Oliver S (2013). Using qualitative evidence on patients’ views to help understand variation in effectiveness of complex interventions: a qualitative comparative analysis. Trials.

[10] Wells M, Williams B, Treweek S, Coyle J, Taylor J (2012). Intervention description is not enough: evidence from an in-depth multiple case study on the untold role and impact of context in randomised controlled trials of seven complex interventions. Trials.

[11] Voigt-Radloff S, Graff M, Leonhart R, Hull M, Rikkert MO, Vernooij-Dassen M. Why did an effective Dutch complex psycho-social intervention for people with dementia not work in the German healthcare context? Lessons learnt from a process evaluation alongside a multicentre RCT. BMJ Open. 2011;1:e000094. 10.1136/bmjopen-2011-000094 PMC3191434 22021759

[12] Lewin S, Glenton C, Oxman AD (2009). Use of qualitative methods alongside randomised controlled trials of complex healthcare interventions: methodological study. BMJ.

[13] O’Cathain A, Thomas K, Drabble S, Rudolph A, Goode J, Hewison J. Maximising the value of combining qualitative research and randomised controlled trials in health research: the qualitative research in trials (QUART) study – a mixed methods study. Health Technol Assess. 2014;18. 10.3310/hta18380 PMC4781055 24914457

[14] Burchett HED, Leurent B, Baiden F, Baltzell K, Björkman A, Bruxvoort K, Clarke S, DiLiberto D, Elfving K, Goodman C (2017). Improving prescribing practices with rapid diagnostic tests (RDTs): synthesis of 10 studies to explore reasons for variation in malaria RDT uptake and adherence. BMJ Open.

[15] O’Cathain A, Hoddinott P, Lewin S, Thomas KJ, Young B, Adamson J, Jansen YJ, Mills N, Moore G, Donovan JL (2015). Maximising the impact of qualitative research in feasibility studies for randomised controlled trials: guidance for researchers. Pilot Feasibility Stud.

[16] Hawe P, Shiell A, Riley T, Gold L (2004). Methods for exploring implementation variation and local context within a cluster randomised community intervention trial. J Epidemiol Community Health.

[17] The review on antimicrobial resistance (2016). tackling drug-resistant infections globally: final report and recommendations.

[18] World Bank. 2017. “Drug-Resistant Infections: A Threat to Our Economic Future.” Washington, DC: World Bank. License: Creative Commons Attribution CC BY 3.0 IGO.

[19] Morgan DJ, Okeke IN, Laxminarayan R, Perencevich EN, Weisenberg S (2011). Non-prescription antimicrobial use worldwide: a systematic review. Lancet Infect Dis.

[20] Kumarasamy KK, Toleman MA, Walsh TR, Bagaria J, Butt F, Balakrishnan R, Chaudhary U, Doumith M, Giske CG, Irfan S (2010). Emergence of a new antibiotic resistance mechanism in India, Pakistan, and the UK: a molecular, biological, and epidemiological study. Lancet Infect Dis.

[21] Klein EY, Van Boeckel TP, Martinez EM, Pant S, Gandra S, Levin SA, Goossens H, Laxminarayan R. Global increase and geographic convergence in antibiotic consumption between 2000 and 2015. Proc Natl Acad Sci. 2018;115:E3463-E3470. 10.1073/pnas.1717295115 PMC5899442 29581252

[22] Public Health England. UK case of Neisseria gonorrhoeae with high-level resistance to azithromycin and resistance to ceftriaxone acquired abroad. Health Prot Rep Adv Access Rep. 2018;12.

[23] Butler CC, Hood K, Verheij T, Little P, Melbye H, Nuttall J, Kelly MJ, Mölstad S, Godycki-Cwirko M, Almirall J (2009). Variation in antibiotic prescribing and its impact on recovery in patients with acute cough in primary care: prospective study in 13 countries. BMJ.

[24] Linder JA (2015). Comparative effectiveness of three anxiolytics for acute respiratory infections: antibiotics, C-reactive protein point-of-care testing, and improved communication. J Gen Intern Med.

[25] Hoa NQ, Thi Lan P, Phuc HD, Chuc NTK, Stalsby Lundborg C (2017). Antibiotic prescribing and dispensing for acute respiratory infections in children: effectiveness of a multi-faceted intervention for health-care providers in Vietnam. Glob Health Action.

[26] Phuong NTK, Hoang TT, Van PH, Tu L, Graham SM, Marais BJ (2017). Encouraging rational antibiotic use in childhood pneumonia: a focus on Vietnam and the Western Pacific Region. Pneumonia.

[27] Davey P, Marwick CA, Scott CL, Charani E, McNeil K, Brown E, Gould IM, Ramsay CR, Michie S. Interventions to improve antibiotic prescribing practices for hospital inpatients. Cochrane Database Syst Rev. 2017. 10.1002/14651858.CD003543.pub4 PMC6464541 28178770

[28] Nesta (2018). Longitude prize.

[29] Aabenhus R, Jensen JUS, Jorgensen KJ, Hrobjartsson A, Bjerrum L (2014). Biomarkers as point-of-care tests to guide prescription of antibiotics in patients with acute respiratory infections in primary care. Cochrane Database Syst Rev.

[30] Nora D, Salluh J, Martin-Loeches I, Póvoa P (2017). Biomarker-guided antibiotic therapy—strengths and limitations. Ann Transl Med.

[31] Keitel K, Kagoro F, Samaka J, Masimba J, Said Z, Temba H, Mlaganile T, Sangu W, Rambaud-Althaus C, Gervaix A (2017). A novel electronic algorithm using host biomarker point-of-care tests for the management of febrile illnesses in Tanzanian children (e-POCT): a randomized, controlled non-inferiority trial. PLoS Med.

[32] Lubell Y, Blacksell SD, Dunachie S, Tanganuchitcharnchai A, Althaus T, Watthanaworawit W, Paris DH, Mayxay M, Peto TJ, Dondorp AM (2015). Performance of C-reactive protein and procalcitonin to distinguish viral from bacterial and malarial causes of fever in Southeast Asia. BMC Infect Dis.

[33] Hildenwall H, Muro F, Jansson J, Mtove G, Reyburn H, Amos B (2017). Point-of-care assessment of C-reactive protein and white blood cell count to identify bacterial aetiologies in malaria-negative paediatric fevers in Tanzania. Tropical Med Int Health.

[34] Van den Bruel A, Jones C, Thompson M, Mant D (2016). C-reactive protein point-of-care testing in acutely ill children: a mixed methods study in primary care. Arch Dis Child.

[35] Cals JWL, Schot MJC, de Jong SAM, Dinant G-J, Hopstaken RM (2010). Point-of-care C-reactive protein testing and antibiotic prescribing for respiratory tract infections: a randomized controlled trial. Ann Fam Med.

[36] Cals JWL, de Bock L, Beckers P-JHW, Francis NA, Hopstaken RM, Hood K, de Bont EGPM, Butler CC, Dinant G-J (2013). Enhanced communication skills and C-reactive protein point-of-care testing for respiratory tract infection: 3.5-year follow-up of a cluster randomized trial. Ann Fam Med.

[37] Drain PK, Hyle EP, Noubary F, Freedberg KA, Wilson D, Bishai WR, Rodriguez W, Bassett IV (2014). Diagnostic point-of-care tests in resource-limited settings. Lancet Infect Dis.

[38] Lubell Y, Althaus T (2017). Biomarker tests for bacterial infection-a costly wait for the holy grail. Lancet Infect Dis.

[39] Do NTT, Ta NTD, Tran NTH, Than HM, Vu BTN, Hoang LB, van Doorn HR, Vu DTV, Cals JWL, Chandna A (2016). Point-of-care C-reactive protein testing to reduce inappropriate use of antibiotics for non-severe acute respiratory infections in Vietnamese primary health care: a randomised controlled trial. Lancet Glob Health.

[40] Bazeley P (2013). Qualitative data analysis: practical strategies.

[41] Huddy JR, Ni MZ, Barlow J, Majeed A, Hanna GB (2016). Point-of-care C reactive protein for the diagnosis of lower respiratory tract infection in NHS primary care: a qualitative study of barriers and facilitators to adoption. BMJ Open.

[42] Hardy V, Thompson M, Keppel GA, Alto W, Dirac MA, Neher J, Sanford C, Hornecker J, Cole A (2017). Qualitative study of primary care clinicians’ views on point-of-care testing for C-reactive protein for acute respiratory tract infections in family medicine. BMJ Open.

[43] Gelband H, Miller-Petrie M, Pant S, Gandra S, Levinson J, Barter D, White A, Laxminarayan R (2015). State of the world’s antibiotics, 2015.

[44] Quet F, Vlieghe E, Leyer C, Buisson Y, Newton PN, Naphayvong P, Keoluangkhot V, Chomarat M, Longuet C, Steenkeste N, Jacobs J (2015). Antibiotic prescription behaviours in Lao People’s Democratic Republic: a knowledge, attitude and practice survey. Bull World Health Organ.

[45] Holloway KA, Batmanabane G, Puri M, Tisocki K (2017). Antibiotic use in South East Asia and policies to promote appropriate use: reports from country situational analyses. BMJ.

[46] O’Cathain A (2018). A practical guide to using qualitative research with randomized controlled trials.

[47] Haenssgen MJ, Charoenboon N, Althaus T, Greer RC, Intralawan D, Lubell Y (2018). The social role of C-reactive protein point-of-care testing to guide antibiotic prescription in Northern Thailand. Soc Sci Med.

[48] Do TTN. Assessing and improving rational antimicrobial use in urban and rural health care facilities in Vietnam. PhD Thesis. Milton Keynes: Open University; 2017.

[49] Lapadat JC, Mills AJ, Eurepos G, Wiebe E (2010). Thematic analysis. Encyclopedia of case study research.

[50] Nga do TT, Chuc NT, Hoa NP, Hoa NQ, Nguyen NT, Loan HT, Toan TK, Phuc HD, Horby P, Van Yen N (2014). Antibiotic sales in rural and urban pharmacies in northern Vietnam: an observational study. BMC Pharmacol Toxicol.

[51] Guest G, Bunce A, Johnson L (2006). How many interviews are enough? An experiment with data saturation and variability. Field Methods.

[52] Kerr C, Nixon A, Wild D (2010). Assessing and demonstrating data saturation in qualitative inquiry supporting patient-reported outcomes research. Expert Rev Pharmacoecon Outcomes Res.

[53] Radyowijati A, Haak H (2003). Improving antibiotic use in low-income countries: an overview of evidence on determinants. Soc Sci Med.

[54] Ruizendaal E, Dierickx S, Peeters Grietens K, Schallig HD, Pagnoni F, Mens PF (2014). Success or failure of critical steps in community case management of malaria with rapid diagnostic tests: a systematic review. Malar J.

[55] Sheikh K, Porter JDH, Sheikh K, George A (2010). Understanding practitioners’ responses to national policy guidelines: the case of HIV testing in hospitals. Health providers in India: on the frontlines of change.

[56] Lune H, Berg BL (2017). Qualitative research methods for the social sciences.

[57] QSR International (2017). Nvivo 11.

[58] Lewis J, Ritchie J, Lewis J (2003). Design issues. Qualitative research practice: a guide for social science students and researchers.

[59] Ritchie J, Spencer L, O’Connor W, Ritchie J, Lewis J (2003). Carrying out qualitative analysis. Qualitative research practice: a guide for social science students and researchers.

[60] Minh NNQ. Outpatient antibiotic use in acute respiratory infections in Ho Chi Minh City, Vietnam. PhD Thesis. Milton Keynes: Open University; 2014.

[61] Forbes EI (2016). On the frontier of urbanization: informal settlements in Yangon, Myanmar. Indep J Burmese Scholarsh.

[62] Htwe T, Oo WM, Lwin N, Win KH, Dar HT (2017). Poverty among households living in slum area of Hlaing Tharyar Township, Yangon City, Myanmar. Int J Res Med Sci.

[63] World Bank (2018). World databank.

[64] Sumpradit N, Chongtrakul P, Anuwong K, Pumtong S, Kongsomboon K, Butdeemee P, Khonglormyati J, Chomyong S, Tongyoung P, Losiriwat S (2012). Antibiotics smart use: a workable model for promoting the rational use of medicines in Thailand. Bull World Health Organ.

[65] MPH, MAC (2017). National strategic plan on antimicrobial resistance 2017–2021, Thailand.

[66] Sumpradit N, Wongkongkathep S, Poonpolsup S, Janejai N, Paveenkittiporn W, Boonyarit P, Jaroenpoj S, Kiatying-Angsulee N, Kalpravidh W, Sommanustweechai A, Tangcharoensathien V (2017). New chapter in tackling antimicrobial resistance in Thailand. BMJ.

[67] Tangcharoensathien V, Sattayawutthipong W, Kanjanapimai S, Kanpravidth W, Brown R, Sommanustweechai A (2017). Antimicrobial resistance: from global agenda to national strategic plan, Thailand. Bull World Health Organ.

[68] Nguyen KV, Thi Do NT, Chandna A, Nguyen TV, Pham CV, Doan PM, Nguyen AQ, Thi Nguyen CK, Larsson M, Escalante S (2013). Antibiotic use and resistance in emerging economies: a situation analysis for Viet Nam. BMC Public Health.

[69] Holloway KA (2011). Myanmar: drug policy and pharmaceuticals in health care delivery.

[70] Ministry of Health and Sports (2017). National action plan for containment of antimicrobial resistance: Myanmar, 2017-2022 (draft, Version 01).

[71] Greer RC, Intralawan D, Mukaka M, Wannapinij P, Day NPJ, Nedsuwan S, Lubell Y (2018). Retrospective review of the management of acute infections and the indications for antibiotic prescription in primary care in northern Thailand. BMJ Open.

[72] Apidechkul T, Laingoen O, Suwannaporn S (2016). Inequity in accessing health care service in Thailand in 2015: a case study of the hill tribe people in Mae Fah Luang district, Chiang Rai, Thailand. J Health Res.

[73] Khine Zaw Y, Charoenboon N, Haenssgen MJ, Lubell Y (2018). A comparison of patients’ local conceptions of illness and medicines in the context of C-reactive protein biomarker testing in Chiang Rai and Yangon. Am J Trop Med Hyg.

[74] Beisel U, Umlauf R, Hutchinson E, Chandler CIR (2016). The complexities of simple technologies: re-imagining the role of rapid diagnostic tests in malaria control efforts. Malar J.

[75] Hopkins H, Bruxvoort KJ, Cairns ME, Chandler CIR, Leurent B, Ansah EK, Baiden F, Baltzell KA, Björkman A, Burchett HED (2017). Impact of introduction of rapid diagnostic tests for malaria on antibiotic prescribing: analysis of observational and randomised studies in public and private healthcare settings. BMJ.

[76] Okeke IN, Klugman KP, Bhutta ZA, Duse AG, Jenkins P, O’Brien TF, Pablos-Mendez A, Laxminarayan R (2005). Antimicrobial resistance in developing countries. Part II: strategies for containment. Lancet Infect Dis.

[77] Mendelson M, Balasegaram M, Jinks T, Pulcini C, Sharland M (2017). Antibiotic resistance has a language problem. Nature.

[78] Nichter M, Obermeyer CM (2001). Risk, vulnerability, and harm reduction: preventing STIs in Southeast Asia by antibiotic prophylaxis, a misguided practice. Cultural perspectives on reproductive health.

[79] Karumbi J, Garner P. Directly observed therapy for treating tuberculosis. Cochrane Database Syst Rev. 2015. 10.1002/14651858.CD003343.pub4 PMC4460720 26022367

[80] WHO (2017). Treatment of tuberculosis: guidelines for treatment of drug-susceptible tuberculosis and patient care: 2017 update.

[81] Skinner D, Claassens M (2016). It’s complicated: why do tuberculosis patients not initiate or stay adherent to treatment? A qualitative study from South Africa. BMC Infect Dis.

[82] Sagbakken M, Frich JC, Bjune GA, Porter JD (2013). Ethical aspects of directly observed treatment for tuberculosis: a cross-cultural comparison. BMC Med Ethics.

[83] Engel N, Zeiss R (2014). Situating standards in practices: multi drug-resistant tuberculosis treatment in India. Sci Cult.

[84] Spurling GKP, Del Mar CB, Dooley L, Foxlee R, Farley R. Delayed antibiotic prescriptions for respiratory infections. Cochrane Database Syst Rev. 2017;9. 10.1002/14651858.CD004417.pub5 PMC6372405 28881007

[85] Dupas P (2011). Health behavior in developing countries. Annu Rev Econ.

[86] Lau J, Ioannidis JPA, Schmid CH (1998). Summing up evidence: one answer is not always enough. Lancet.

[87] Kravitz RL, Duan N, Braslow J (2004). Evidence-based medicine, heterogeneity of treatment effects, and the trouble with averages. Milbank Q.

[88] Kent DM, Rothwell PM, Ioannidis JP, Altman DG, Hayward RA (2010). Assessing and reporting heterogeneity in treatment effects in clinical trials: a proposal. Trials.

[89] Supakankunti S, Janjaroen WS, Tangphao O, Ratanawijitrasin S, Kraipornsak P, Pradithavanij P (2001). Impact of the World Trade Organization TRIPS Agreement on the pharmaceutical industry in Thailand. Bull World Health Organ.

[90] Thomas C (2002). Trade policy and the politics of access to drugs. Third World Q.

[91] WHO (2007). Strengthening health systems to improve health outcomes: WHO’s framework for action.

