# Supplementary material for: How context can impact clinical trials: a multi-country qualitative case study comparison of diagnostic biomarker test interventions
Source: Trials. 2019 Feb 8;20:111. doi: 10.1186/s13063-019-3215-9 (PMC6368827; doi:10.1186/s13063-019-3215-9)
Supplement: Supplementary file 1 — Case study overview. (DOCX 114 kb) [file 13063_2019_3215_MOESM1_ESM.docx]

# How Context can Impact Clinical Trials: A Multi-Country Qualitative Case Study Comparison of Diagnostic Biomarker Test Interventions

# Supplementary Material

# Case Study Overview

Our study took place at the primary healthcare level in Myanmar, Thailand, and Vietnam. We outline in this section the characteristics of these three countries together with their heterogeneous health policy environment, their fragmented health systems, and the mixed health service utilisation among the target populations. The subsequent Section 3.2 will present the results of our qualitative analysis.

An overview of development and public health indicators from the three country cases is presented in Table 2. Whereas Thailand was classified as a higher-middle income country and Vietnam and Myanmar as lower-middle income countries [[1](#_ENREF_1)], Vietnam compared more favourably to Thailand in terms of development indicators like poverty rate and adult literacy. Myanmar exhibited the poorest record among the three countries, also from a public health perspective. It had comparatively poor performance in health indicators like life expectancy (66 years; Thailand: 75 years; Vietnam: 76 years) and child mortality (51 deaths per 1,000 live births; Thailand: 12; Vietnam: 22). Myanmar’s public health expenditure was also the lowest in the comparison group: Adjusted for local price levels (or purchasing power parity, PPP), its total per capita health expenditure of $103 was only a fraction of health expenditure in Vietnam ($390) and Thailand ($600). With $52 per capita, more than 50% of health expenditure in Myanmar was out-of-pocket spending, compared to 38% in Vietnam and 12% in Thailand (this may be explained by the lack of universal health care and health insurance schemes in Myanmar, as opposed to common health insurance coverage in Vietnam and a universal healthcare policy in Thailand [[2](#_ENREF_2)]).

*Table 2. Country Comparison: Development and Health Indicators*

|  | **Thailand** | **Vietnam** | **Myanmar** |
| --- | --- | --- | --- |
| **GDP per Capita (US$ PPP)** | $15,683 (2016) | $5,838 (2016) | $5,305 (2016) |
| **Poverty Rate  (US$1.90/Day, PPP)** | 0% (2013) | 3% (2014) | 7% (2015) |
| **Literacy rate  (% of Adult Population)** | 93% (2015) | 94% (2009) | 76% (2016) |
| **Access to Improved Sanitation  (% of Population)** | 93% (2015) | 78% (2015) | 80% (2015) |
| **Total Health Expenditure  (US$ PPP)** | $600 (2014) | $390 (2014) | $103 (2014) |
| **Out-of-Pocket Health Expenditure  (US$ PPP)** | $72 (2014) | $144 (2014) | $52 (2014) |
| **Life Expectancy at Birth (Years)** | 75 (2015) | 76 (2015) | 66 (2015) |
| **Under-5 Mortality Rate (per 1,000 live births)** | 12 (2016) | 22 (2016) | 51 (2016) |

Source: [World Bank [1](#_ENREF_1)].

*Notes:* Values in parentheses are year of latest available data. GDP is “gross domestic product;” PPP is “purchasing power parity.”

From a health policy perspective, Thailand exhibited a regionally more advanced state of antimicrobial resistance policies, for example with its 2007 *Antibiotic Smart Use* campaign and its *2017-2021 National Strategic Plan on Antimicrobial Resistance* that aimed at reducing antibiotic prescriptions by 20% by 2021 [[3-6](#_ENREF_3)]. Among others, this AMR policy environment had transpired into health centre operations through active monitoring of antibiotic prescriptions, and it had even inspired local antibiotic-related initiatives by health centres like information leaflets [[7](#_ENREF_7)]. Vietnam, too, had AMR-related policies, the most important of which was the *2005 Drug Law*, intended to improve appropriate antimicrobial use by permitting antimicrobials to be dispensed only with a prescription (Regulation No. 04/2008/QD-BYT and 2016 Circular No. 05/2016/TT-BYT). Yet, unlike the Thai policy environment, the Vietnamese AMR policies were not enforced and did not involve sanctions for non-compliance [[8](#_ENREF_8), [9](#_ENREF_9)]. In contrast to both Thailand and Vietnam, Myanmar had no national strategy to target antimicrobial resistance [[10](#_ENREF_10), [11](#_ENREF_11)] – however, by the time of this research, a *National Action Plan for Containment of Antimicrobial Resistance* was being drafted and steps towards antibiotic regulation were being initiated [[12](#_ENREF_12)].

Despite variations in AMR-related policies and their enforcement, fragmented health systems with extensive private and informal healthcare sectors enabled easy over-the-counter access to antibiotics in all three countries [[8](#_ENREF_8), [11](#_ENREF_11), [13-15](#_ENREF_13)]. The primary healthcare care facilities involved in the clinical trials thereby represented only a fraction of the available healthcare providers in all three settings. Mirroring high degrees of health system fragmentation across low- and middle-income countries [[16](#_ENREF_16)], public healthcare was provided alongside private hospitals and doctors, pharmacies with varying degrees of qualification, and local grocery shops that also sold antibiotics. The utilisation of the various healthcare providers was shaped by the local socio-economic context of our field sites:

- **Chiang Rai** (a northern Thai province bordering Myanmar and Lao PDR): The primary care centres were located in the periphery of the provincial capital city, but their catchment population extended from peri-urban citizens to rural unregistered members of ethnic minority groups [[17](#_ENREF_17)]. Because public healthcare in Thailand was free only for the registered population, undocumented parts of the population could still incur out-of-pocket expenditure for health service access, and language differences between minority groups and public healthcare providers could create further frictions in the patient-provider relationship [[7](#_ENREF_7)]. Public healthcare was commonly accessed by poorer segments of the population, provided that these facilities were neither overcrowded or out of reach—in which case private and informal healthcare providers became the first choice.
- **Yangon** (Myanmar’s largest and former capital city): The study clinics were located in sub-urban slum areas (primarily Hlaing Tha Yar, Yangon’s poorest and most populated district), which were characterised by poverty rates as high as 59%, a high share of squatter populations, and a high incidence of diseases related to poor hygiene and environmental conditions (i.e. diarrhoea, dysentery, malaria, and tuberculosis) [[18](#_ENREF_18), [19](#_ENREF_19)]. While the public hospital participating in the trial provided comprehensive primary healthcare, the three non-governmental organisation (NGO) clinics specialised in maternal and child health care, sexually transmitted infections, HIV, and tuberculosis. Patient management including antibiotic prescription was free of charge in all the facilities. Alternative common channels to access antibiotics and other medicine were shops and pharmacies, which were widespread in the local study area and often offered unlabelled medicine sets (so-called “drug cocktails”) [[14](#_ENREF_14)].
- **Hanoi** (Vietnam’s capital and its second most populous city): Nine out of the ten primary care facilities in the clinical trial were urban policlinics in Hanoi, and one rural facility comprised outpatient departments of a rural hospital 60km west of Hanoi. The health system comprised approximately 35 private clinics and 50 private pharmacies for each of the 1,000 public hospitals in Vietnam [[20](#_ENREF_20)]. Although healthcare was not free and health insurance coverage was still incomplete, the dense network of public and private healthcare providers had led to improvements in the quality and accessibility of healthcare, but it had also resulted in an increase of antibiotics consumption [[20](#_ENREF_20), [21](#_ENREF_21)]. Community-level public primary care services were thereby more commonly accessed by the poor and people with health insurance, but the first and cheaper step during an illness was typically self-medication [[9](#_ENREF_9), [20](#_ENREF_20)].

In summary, Thailand was a regional AMR policy leader and its health and development indicators compared favourably against Myanmar and Vietnam. Variations in universal healthcare coverage meant that out-of-pocket expenditures for healthcare were high in Vietnam and Myanmar. However, healthcare systems remained fragmented and low extreme poverty rates masked persistent constraints and inequities in healthcare access for sub-populations within all three field sites.

# References

1. World Bank: **World databank.** 2018. <http://databank.worldbank.org/data/home.aspx>. Accessed October 4, 2018

2. Jongudomsuk P, Srithamrongsawat S, Patcharanarumol W, Limwattananon S, Pannarunothai S, Vapatanavong P, Sawaengdee K, Fahamnuaypol P: **The Kingdom of Thailand health system review.** In *Health Systems in Transition.* *Volume* 5, No. 5. Edited by Tangcharoensathien V. Manila: Asia Pacific Observatory on Health Systems and Policies; 2015

3. Sumpradit N, Chongtrakul P, Anuwong K, Pumtong S, Kongsomboon K, Butdeemee P, Khonglormyati J, Chomyong S, Tongyoung P, Losiriwat S, et al: **Antibiotics Smart Use: a workable model for promoting the rational use of medicines in Thailand.** *Bulletin of the World Health Organization* 2012, **90:**905-13.

4. MPH, MAC: **National strategic plan on antimicrobial resistance 2017-2021, Thailand.** Nonthaburi: Ministry of Public Health and Ministry of Agriculture and Cooperatives; 2017.

5. Sumpradit N, Wongkongkathep S, Poonpolsup S, Janejai N, Paveenkittiporn W, Boonyarit P, Jaroenpoj S, Kiatying-Angsulee N, Kalpravidh W, Sommanustweechai A, Tangcharoensathien V: **New chapter in tackling antimicrobial resistance in Thailand.** *BMJ* 2017, **358**.

6. Tangcharoensathien V, Sattayawutthipong W, Kanjanapimai S, Kanpravidth W, Brown R, Sommanustweechai A: **Antimicrobial resistance: from global agenda to national strategic plan, Thailand.** *Bulletin of the World Health Organization* 2017, **95:**599-603.

7. Haenssgen MJ, Charoenboon N, Althaus T, Greer RC, Intralawan D, Lubell Y: **The social role of C-reactive protein point-of-care testing to guide antibiotic prescription in Northern Thailand.** *Social Science & Medicine* 2018, **202:**1-12.

8. Nga do TT, Chuc NT, Hoa NP, Hoa NQ, Nguyen NT, Loan HT, Toan TK, Phuc HD, Horby P, Van Yen N, et al: **Antibiotic sales in rural and urban pharmacies in northern Vietnam: an observational study.** *BMC Pharmacology and Toxicology* 2014, **15**.

9. Nguyen KV, Thi Do NT, Chandna A, Nguyen TV, Pham CV, Doan PM, Nguyen AQ, Thi Nguyen CK, Larsson M, Escalante S, et al: **Antibiotic use and resistance in emerging economies: a situation analysis for Viet Nam.** *BMC Public Health* 2013, **13**.

10. Holloway KA: **Myanmar: drug policy and pharmaceuticals in health care delivery.** New Delhi: World Health Organization Regional Officer for South East Asia; 2011.

11. Holloway KA, Batmanabane G, Puri M, Tisocki K: **Antibiotic use in South East Asia and policies to promote appropriate use: reports from country situational analyses.** *BMJ* 2017, **358**.

12. Ministry of Health and Sports: **National action plan for containment of antimicrobial resistance: Myanmar, 2017-2022 (draft, Version 01).** Naypyidaw: Ministry of Health and Sports; 2017.

13. Apisarnthanarak A, Mundy L: **Comparison of methods of measuring pharmacy sales of antibiotics without prescriptions in Pratumthani, Thailand.** *Infection Control and Hospital Epidemiology* 2009, **30:**1130-2.

14. Khine Zaw Y, Charoenboon N, Haenssgen MJ, Lubell Y: **A comparison of patients’ local conceptions of illness and medicines in the context of C-reactive protein biomarker testing in Chiang Rai and Yangon.** *American Journal of Tropical Medicine and Hygiene* 2018, **98:**1661-760.

15. Khamsarn S, Nampoonsak Y, Busamaro S, Tangkoskul T, Seenama C, Rattanaumpawan P, Boonyasiri A, Thamlikitkul V: **Epidemiology of antibiotic use and antimicrobial resistance in selected communities in Thailand.** *Journal of the Medical Association of Thailand* 2016, **99:**270-5.

16. Sudhinaraset M, Ingram M, Lofthouse HK, Montagu D: **What is the role of informal healthcare providers in developing countries? A systematic review.** *PLoS ONE* 2013, **8:**e54978.

17. Apidechkul T, Laingoen O, Suwannaporn S: **Inequity in accessing health care service in Thailand in 2015: a case study of the hill tribe people in Mae Fah Luang district, Chiang Rai, Thailand.** *Journal of Health Research* 2016, **30:**67-71.

18. Forbes EI: **On the frontier of urbanization: informal settlements in Yangon, Myanmar.** *Independent Journal of Burmese Scholarship* 2016, **1:**197-238.

19. Htwe T, Oo WM, Lwin N, Win KH, Dar HT: **Poverty among households living in slum area of Hlaing Tharyar Township, Yangon City, Myanmar.** *International Journal of Research in Medical Sciences* 2017, **5:**2497-501.

20. Do TTN: **Assessing and improving rational antimicrobial use in urban and rural health care facilities in Vietnam.** *PhD Thesis.* Open University, 2017.

21. Tran VT, Hoang TP, Nguyen TKP: **A health financing review of Viet Nam with a focus on social health insurance: bottlenecks in instutional design and organizational practice of health financing and options to accelerate progress towards universal coverage.** Geneva: World Health Organization; 2011.
